# 134. Yield of Fungal and Mycobacterial Cultures for Operative Specimens in Orthopedic Procedures

**DOI:** 10.1093/ofid/ofad500.207

**Published:** 2023-11-27

**Authors:** Margaret Cooper, Katherine C Shihadeh, Michael Deaney, Whitney Miller, Joshua Parry, Michael L Wilson, Timothy C Jenkins

**Affiliations:** Denver Health Medical Center, Denver, Colorado; Denver Health, Denver, Colorado; Denver Health Medical Center, Denver, Colorado; Denver Health, Denver, Colorado; Denver Health Medical Center, Denver, Colorado; Denver Health and Hospital Authority, Denver, Colorado; Denver Health and Hospital Authority, Denver, Colorado

## Abstract

**Background:**

Fungal and mycobacterial cultures are routinely sent on orthopedic operative specimens, but their impact on clinical care is not well established. Processing fungal and mycobacterial cultures is time and labor intensive, associated with significant healthcare costs, and may yield false-positive results due to contamination. The objective of this study was to determine the utility and diagnostic yield of fungal and mycobacterial cultures for orthopedic infections.

**Methods:**

Patients undergoing surgery by the orthopedic or podiatry service that had operative specimens sent for fungal and mycobacterial cultures from January through December 2022 were included. Descriptive statistics were used to evaluate yield of fungal and mycobacterial cultures and incremental yield of fungal cultures over standard bacterial culture media. A random subset of patients was manually reviewed to determine whether microorganisms identified by fungal or mycobacterial cultures were considered clinically relevant and whether culture results changed management.

**Results:**

Fungal and mycobacterial cultures were ordered on a total of 1148 operative specimens in 428 patients. A mean of 2.1 ± 1.4 specimens was collected per operative procedure. A microorganism was identified in 33 (2.8%) of fungal cultures and in no mycobacterial cultures. Fungal organisms included yeast (90.9%) and mold (9.1%).

Of 50 randomly selected patients, a total of 249 operative specimens were collected (mean 5 per patient, 2.7 per operative procedure). A microorganism was identified by fungal culture in 5 (2%) specimens from 3 patients. All three patients were treated with antifungal agents indicating the fungi were deemed to be clinically relevant. However, the fungi also grew on the standard bacterial media from all 5 specimens suggesting there was no incremental benefit of the fungal cultures. There were no positive mycobacterial cultures, and no patients received mycobacterial treatment.

**Conclusion:**

The diagnostic yield of fungal and mycobacterial cultures on operative specimens in orthopedic and podiatry surgery is very low and rarely results in a change in therapeutic management. The results of this study demonstrate an opportunity for diagnostic stewardship for these procedures.

**Disclosures:**

**Timothy C. Jenkins, MD**, Basilea: Clinical events adjudication committee

